# Surgical outcomes of nephrectomy for elderly patients with renal cell carcinoma

**DOI:** 10.12669/pjms.342.14062

**Published:** 2018

**Authors:** Xiaomin Gao, Liang Hu, Yue Pan, Lei Zheng

**Affiliations:** 1 Xiaomin Gao, Department of Urology, The Third Clinical Institute Affiliated to Wenzhou Medical University, The People’s Hospital of Wenzhou, Wenzhou, China; 2 Liang Hu, Department of Urology, Jinhua Center Hospital, Jinhua, China; 3 Yue Pan, Department of Urology, The First Affiliated Hospital of Wenzhou Medical University, Wenzhou, China; 4 Lei Zheng, Department of Urology, The Third Clinical Institute Affiliated to Wenzhou Medical University, The People’s Hospital of Wenzhou, Wenzhou, China

**Keywords:** Age groups, Nephrectomy, Renal cell carcinoma, Survival

## Abstract

**Objective:**

The feasibility of curative surgery for elderly patients with renal cell carcinoma (RCC) remains controversial and under discussion. The main aim of this study was to evaluate the long-term benefits of curative surgery as a treatment for RCC in elderly patients.

**Methods:**

We retrospectively considered 672 patients with RCC who underwent partial nephrectomy or radical nephrectomy between January 2004 and July 2014. X-tile program was used to determine the optimal age cutoff values with CSS as endpoint.

**Results:**

Patients were divided into the following groups according to their age using the method of X-tile program: a young group (< 40 years), a young-old group (40-75) and an old-old group (≥ 75). Following multivariate analysis age ≥ 75 years was determined to be an independent risk factor for overall survival (HR=4.36; 95% CI: 1.31-14.48; P=0.016); interestingly, this was not the case for cancer-specific survival (HR = 2.65; 95%CI: 0.77-9.16; P=0.124). Furthermore, an age of 40 to 75 years was not a risk factor according to univariate and multivariate analysis.

**Conclusion:**

After determining the age cutoff values, there was no significant difference in prognosis between young and old patients with RCC.

## INTRODUCTION

Renal cell carcinoma (RCC) is well-known as the most common renal parenchymal malignancy, representing 2% to 3% of all cancers.^1^ In 2013, more than 350, 000 cases and 140, 000 deaths from RCC occurred worldwide.^2^ Like other common genitourinary carcinomas, RCC typically affects older patients; in fact, only 5% of all RCC patients are younger than 40 years old and the median age at diagnosis remains between 60 and 65 years.^3^,^4^ The incidence of RCC varies worldwide; notably, as the overall Chinese population ages and the average Chinese lifespan gradually increases,^5^,^6^ the number of elderly patients in China who have been diagnosed with malignancy continues to increase. In spite of the advances that have been made in the understanding of RCC biology, surgery procedures, including radical nephrectomy or partial nephrectomy, remain the only curative treatment method for localized RCC patients.^1^,^7^ Older patients are generally considered to have decreased reserve capacity and a higher risk and incidence of adverse outcomes after surgery. Therefore, surgeons often hesitate to perform surgery for elderly RCC patients, due the high frequency of mortality and complications.^8^-^11^

Recently, several studies have been completed that have evaluated the long-term benefits of curative surgery in old patients with non-metastatic RCC. Focusing on these individuals’ long-term survival, some studies have reported that there was no notable statistical difference in prognosis between young and old patients with RCC^4^,^12^,^13^; these authors found that elderly patients could benefit from the curative surgery, and yielded cancer-specific survival (CSS) rates similar to those of young patients. On the other hand, there have been other studies that have emphasized that elderly patients had worse CSS rates compared with young patients.^10^,^14^-^16^ Therefore, the feasibility of curative surgery for elderly patients with RCC remains controversial and under discussion. Furthermore, the patient ages in these studies were inconsistent, with ranges of 40-79^13^, 60-70^4^, 60-80^12^, and ≥ 75^14^ years considered, respectively.

In this retrospective study, we evaluate the clinicopathological features and survival outcomes of patients undergoing curative nephrectomy at different ages, in an attempt to find possible associated factors and appropriate age cut-off values to predict the prognosis of RCC patients after surgery.

## METHODS

### Study design and population

Consecutive RCC patients who underwent radical or partial nephrectomy from January 2004 to July 2014 at the Urologic Department of The First Affiliated Hospital of Wenzhou Medical University, China, were included in this study. This study was approved by the ethics committee of The First Affiliated Hospital of Wenzhou Medical University.

Clinical data, including clinicopathologic and hematologic records, were collected and retrospectively analyzed. Overall survival (OS) and CSS were calculated from the date of surgery to the date of all-cause death, cancer-specific death, or the last follow-up date, respectively. Information on the occurrence of death was obtained from a telephone interview, outpatient medical records, or the patient’s social security death index. The primary endpoint of this study was CSS, and the follow-up cutoff was September 1, 2016.

### Statistical analysis

X-tile program (Version 3.6.1, Yale University, New Haven, CT, USA) was used to determine the optimal age values. The continuous data that were subjected to normal distribution were presented as mean ± standard deviation, and the non-normally distributed data were estimated as median and interquartile range (IQR). Two sample independent *t* tests (Mann-Whitney *U*-test for non-normally distributed data) and chi-square test or Fisher’s exact test were used to test for differences in continuous and categorical variables, respectively. Kaplan-Meier survival curves with log-rank tests and Cox proportional hazard regression analyses were used to compare the OS and CSS rates. Variables with p<0.05 in the univariate Cox regression analysis were included in the multivariate analysis. All tests were two-sided, and differences were considered statistically significant at p<0.05. Statistical analyses were performed using the SPSS software package version 22.0 (IBM Corp., Armonk, NY, USA).

## RESULTS

### Grouping

**Fig.1:**
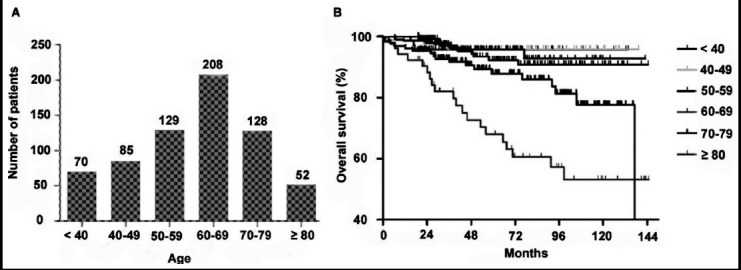
(A-B) The variations of patients number and overall survival rates with advancing age after surgery. Supporting information: Fig.S1 X-tile analyses of cancer-specific survival were performed using patients' data to determine the optimal cutoff values for age. The sample of RCC patients were divided into two groups according to cutoff value. X-tile plot of younger group was shown in the left panels. The optimal cutoff value highlighted by the white circle in the left panels are shown in histograms of the entire cohort (middle panels), and the Kaplan-Meier plots are displayed in the right panels.

From January 2004 to July 2014, a total of 672 patients were included in our study. These patients were divided into 6 groups according to their age distribution (< 40, 40-49, 50-59, 60-69, 70-79, ≥ 80 years, respectively). According to Fig.1 A, B, the OS rate decreased with advancing age; sharp and slight variations were observed at 75 years using the X-tile program (Fig.S1). The χ^2^ log-rank value of the age was 23.2087. The age of 40 years was used to discriminate between young and old patients in most previous studies; therefore, the patients in the current study were divided into three groups: a young group (< 40 years), a young-old group (40-75) and an old-old group (≥ 75).

Among the 672 patients with RCC, 70 (10.4%), 490 (72.9%), and 112 (16.7%) were aged < 40, 40-75, and ≥ 75 years old, respectively (Fig.1). These patients’ clinical and pathological features are detailed in Table-I. There were no significant differences in terms of patient sex, tumor size, pathological T stage, and tumor grade among the three groups. However, ASA grade III (16.3%, 5.25, and 1.4%, respectively; *p*< 0.001) and lower BMI (22.3, 23.3, and 23.4, respectively; *p* = 0.009) were more common in the old-old group than in the other two groups. Although the tumor size was comparable among the three age groups (4.0, 4.3, and 4.3, respectively; p=0.172), patients aged ≥ 75 years old were more likely to undergo radical nephrectomy, rather than partial nephrectomy, as compared with young-old group or young group patients (85.4%, 76.8%, and 68.6%, respectively; p=0.021). Additionally, there were significant differences in histologic subtype among the non-metastatic RCC patients according to age (p=0.001). Specifically, patients in the young-old or old-old groups were diagnosed with clear cell RCC more often than patients in the young group (88.5%, 88.6%, and 75.7%, respectively; p=0.010). In contrast, patients in the young group were more likely to have chromophobe histology as compared with patients in the young-old or old-old groups (17.1%, 5.4%, and 4.1%, respectively; p< 0.001).

**Table-I:** Patient demographic and clinical characteristics.

Factors	Young group	Young-old group	Old-old group	P-value

	N = 70	N = 479	N = 123	
Age (SD)	33.1 (2.9)	60.2 (7.4)	80.0 (4.6)	< 0.001
*Sex*				0.647
Female	25 (35.7%)	154 (32.3%)	36 (29.3%)	
Male	45 (64.3%)	325 (67.8%)	87 (70.7%)	
*ASA grade*				< 0.001
I	17 (24.3%)	80 (16.7%)	6 (4.9%)	
II	52 (74.3%)	374 (78.1%)	97 (78.9%)	
III	1 (1.4%)	25 (5.2%)	20 (16.3%)	
BMI (SD)	23.4 (3.5)	23.2 (3.0)	22.3 (2.7)	0.009
*Type of surgery*				0.021
Partial nephrectomy	22 (31.4%)	111 (23.2%)	18 (14.6%)	
Radical nephrectomy	48 (68.6%)	368 (76.8%)	105 (85.4%)	
Mean tumor size (IQR)	4.0 (3.0)	4.3 (2.5)	4.3 (2.5)	0.172
*Pathological T stage*				0.493
pT1	50 (71.4%)	349 (72.9%)	89 (72.4%)	
pT2	10 (14.3%)	62 (12.9%)	9 (7.3%)	
pT3	9 (12.9%)	62 (12.9%)	23 (18.7%)	
pT4	1 (1.4%)	6 (1.3%)	2 (1.6%)	
*Fuhrman grade*				0.132
1	20 (28.6%)	138 (28.8%)	37 (30.1%)	
2	32 (45.7%)	203 (43.4%)	43 (34.9%)	
3	18 (25.7%)	124 (25.9%)	35 (28.5%)	
4	0	14 (2.9%)	8 (6.5%)	
*Histologic subtype*				0.001
Clear cell	53 (75.7%)	424 (88.5%)	109 (88.6%)	
Papillary	3 (4.3%)	28 (5.8%)	8 (6.5%)	
Chromophobe	12 (17.1%)	26 (5.4%)	5 (4.1%)	
Collecting duct	0	1 (0.2%)	0	
Unclassified	2 (2.9%)	0	1 (0.8%)	

**Fig.2:**
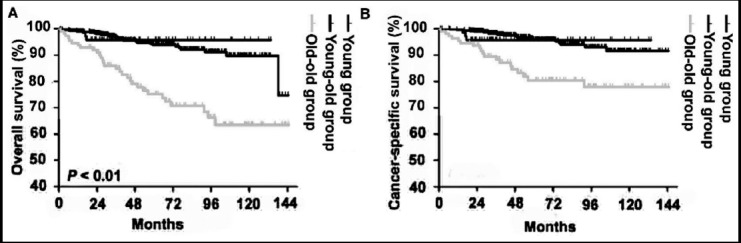
(A-B) Kaplan-Meier survival curves for overall survival and cancer-specific survival rates of the three age groups (young, young-old, old-old groups).

During the follow-up period, 61 patients (9.1%) died, with 41 of them (6.1%) dying from cancer-specific causes within 10 years of follow-up. The median follow up duration was 50.8 months (IQR 30.4-86.1, mean 59.6). As shown in Fig.2, patients aged ≥ 75 years old had a poorer OS and CSS than patients aged < 40 years old or patients aged 40-75 years old (p<0.001); however, the OS (p=0.808) and CSS (p=0.773) rates were comparable between patients aged < 40 years old and patients aged 40-75 years old. The 5-year OS and CSS rates were 95.7% and 95.7%, respectively, for patients in the young group patients; 94.0% and 95.7%, respectively, for patients in the young-old group patients; and 76.0% and 81.8%, respectively, for patients in the old-old group. Table-II shows the results of univariate and multivariate analysis of factors correlated with OS and CSS. Age (≥ 75 years vs. < 40 years) was an independent risk factor for OS (HR = 4.36; 95% CI: 1.31-14.48; P=0.016), but was not significantly associated with death from RCC (HR=2.65; 95% CI: 0.77-9.16; P=0.124). Moreover, BMI, tumor size, T stage, and tumor grade were also independent predictors for OS and CSS.

**Table-II:** Univariate and multivariate logistic regression analysis of risk factors for OS and CSS.

OS	Univariate analysis	Multivariate analysis

	HR (95% CI), P value	HR (95% CI), P value
Sex (male)	1.59 (0.91-2.78), 0.107	
*Age*		
Young-old/Young	1.19 (0.36-3.93), 0.775	1.24 (0.37-4.09), 0.730
Old-old/Young	5.46 (1.67-17.84), 0.005	4.36 (1.31-14.48), 0.016
ASA grade (≥ III)	3.31 (1.75-6.25), < 0.001	1.70 (0.87-3.31), 0.122
BMI (≥ 25)	0.28 (0.11-0.69), 0.006	0.32 (0.13-0.81), 0.016
Type of surgery (Partial nephrectomy)	0.50 (0.21-1.16), 0.106	
Mean tumor size (≥ 7)	2.85 (1.69-4.81), < 0.001	2.12 (1.21-3.72), 0.009
Pathological T stage (≥ 3)	4.04 (2.28-7.18), < 0.001	2.73 (1.47-5.07), 0.002
Fuhrman grade (≥ 3)	2.92 (1.76-4.83), < 0.001	2.12 (1.27-3.54), 0.004
Histologic subtype (Clear cell)	1.07 (0.69-1.67), 0.766	
CSS	Univariate analysis	Multivariate analysis
	HR (95% CI), P value	HR (95% CI), P value
Sex (male)	0.97 (0.52-1.82), 0.927	
Age (≥ 65)		
Young-old/Young	0.84 (0.25-2.86), 0.782	0.86 (0.25-2.93), 0.807
Old-old/Young	3.58 (1.06-12.04), 0.040	2.65 (0.77-9.16), 0.124
ASA grade (≥ III)	3.43 (1.58-7.43), 0.002	1.82 (0.80-4.13), 0.153
BMI (≥ 25)	0.36 (0.11-1.15), 0.037	0.42 (0.15-1.19), 0.101
Type of surgery (Partial nephrectomy)	0.36 (0.11-1.15), 0.085	0.67 (0.20-2.26), 0.521
Mean tumor size (≥ 7)	4.33 (2.34-8.00), < 0.001	2.85 (1.46-5.56), 0.002
Pathological T stage (≥ 3)	5.37 (2.77-10.39), < 0.001	3.14 (1.53-6.44), 0.002
Fuhrman grade (≥ 3)	3.79 (2.03-7.07), < 0.001	2.58 (1.37-4.87), 0.003
Histologic subtype (Clear cell)	1.09 (0.65-1.82), 0.749	

## DISCUSSION

The incidence of elderly patients diagnosed with RCC has significantly increased worldwide in the past few decades, primary due to overall population aging; and in many countries, this is a known major public health problem whose resolution is considered urgent. However, though several studies have reported on the association between age and prognosis in RCC,^4^,^12^-^14^ there is still some controversy among experts with regards to the specifics of the relationship. This contention may be due to the inconsistent age classifications utilized in different studies examining patients with RCC (specifically, 40-79, 60-70, 60-80, ≥ 75 years). Therefore, unlike what was done in previous studies, in this study, we evaluated the outcomes of RCC patients after surgery as the first step in stratifying them, and then compared their clinicopathological characteristics, which allowed us to identify significant differences.

Previous studies have demonstrated that patients aged < 40 years old with other malignancies such as breast cancer generally have worse CSS after surgery as compared with older patients with the same.^17^,^18^ However, our results do not show this in the case of RCC patients. Although older patients were found to have significant poorer OS rates after surgery than those < 40 years of age, there was no statistical difference with respect to CSS between these two groups on multivariate analysis. The results of our study are comparable to those of previous reports. The Mayo Clinic previously conducted a retrospective review from 1970 to 2000, in which they evaluated the CSS rates of 124 patients aged 18 to 40 years old and 1067 patients aged 60 to 70 years old.^4^ Their results revealed that no statistical significance was observed in these two groups. Thompson et al. also described the results of a study in which 1,720 RCC patients were enrolled from 1989 to 2005 and divided into three age groups: < 40 years, 40-59 years, and 60-79 years. The authors reported that they did not observe a significant difference in CSS according to age.^12^ Recently, Kim et al. investigated the influence of age at diagnosis on CSS in RCC, and performed propensity score matching to adjust for potential baseline confounders.^13^ Their data showed that young RCC patients presented with better CSS than older patients did before the two groups were matched, and that the CSS appeared to be similar in the matched cohort. This study employed minimum inherent selection using the analysis of propensity score matching that may help to address the controversy on the feasibility of curative surgery for old patients. In our study, we performed an investigation using the method of X-tile program to identify significant differences. However, patients in the old-old group patients still failed to demonstrate a significantly poorer CSS than patients in the young group. Therefore, considering the worse OS and similar CSS rates of older RCC patients that are present following curative surgery, the selection of surgery candidates should not merely be based on chronological age, but should also consider the patient’s life expectancy and functional status. When considering the confounding factors, young-old age was not a risk predictor for OS and CSS rates, indicating that patients aged 40-75 years old would likely receive the same benefit from surgery as patients aged < 40 years old.

Therefore, in this setting, two hypotheses may be considered. First, according to the world Health Organization’s 2014 World Health Report, the average life expectancy of Chinese individuals has sharply increased during the past decade.[6]^6^ We speculate that CSS rates have improved in older patients because most of them have lived long enough to benefit from surgery. Second, should the selection of candidates who will be treated with nephrectomy take only their life expectancy and functional status into consideration, despite them being elderly? In other words, should age not be considered as important in estimating the prognosis of RCC patients after surgery? Unfortunately, no current study can reliably examine such hypotheses, and it remains to be tested in a prospective randomized trial. However, our results do suggest the necessity for older patients to be treated with nephrectomy to avoid the potential risk.

There are several limitations in the present study. First, this study was conducted at a single center and was retrospective in nature. However, we believe our data to be representative and reliable because our department is the largest urologic cancer center with the largest sample size for RCC patients in the southern part of Zhejiang Province. Second, the sample size of the young-old group and old-old group was relatively small, and the number of deaths recorded was small, possibly in part because of the short follow-up period employed. Both of these may led to a relatively high survival rate in our reported results. Third, cancer-specific mortality rate can be a confounding factor because elderly patients can die due to reasons other than cancer progression. Therefore, this needed to be taken into consideration when comparing the three age groups, and all the authors agree that it is one of the limitations of this study. In conclusion, our results suggest that there was no significant difference in prognosis between young and old patients with RCC.
